# Experimental demonstration of quantum digital signatures over 43 dB channel loss using differential phase shift quantum key distribution

**DOI:** 10.1038/s41598-017-03401-9

**Published:** 2017-06-12

**Authors:** Robert J. Collins, Ryan Amiri, Mikio Fujiwara, Toshimori Honjo, Kaoru Shimizu, Kiyoshi Tamaki, Masahiro Takeoka, Masahide Sasaki, Erika Andersson, Gerald S. Buller

**Affiliations:** 1 Institute of Photonics & Quantum Sciences, and the Scottish Universities Physics Alliance, David Brewster Building, Gait 2, Heriot-Watt University, Edinburgh, EH14 4AS United Kingdom; 2 Quantum ICT Laboratory, National Institute of Information and Communications Technology (NICT), 4-2-1 Nukui Kitamachi, Koganei, Tokyo 184-8795 Japan; 3 NTT Basic Research Laboratories, NTT Corporation, 3-11 Morinosato Wakamiya, Atsugi, Kanagawa 180-8585 Japan

## Abstract

Ensuring the integrity and transferability of digital messages is an important challenge in modern communications. Although purely mathematical approaches exist, they usually rely on the computational complexity of certain functions, in which case there is no guarantee of long-term security. Alternatively, quantum digital signatures offer security guaranteed by the physical laws of quantum mechanics. Prior experimental demonstrations of quantum digital signatures in optical fiber have typically been limited to operation over short distances and/or operated in a laboratory environment. Here we report the experimental transmission of quantum digital signatures over channel losses of up to 42.8 ± 1.2 dB in a link comprised of 90 km of installed fiber with additional optical attenuation introduced to simulate longer distances. The channel loss of 42.8 ± 1.2 dB corresponds to an equivalent distance of 134.2 ± 3.8 km and this represents the longest effective distance and highest channel loss that quantum digital signatures have been shown to operate over to date. Our theoretical model indicates that this represents close to the maximum possible channel attenuation for this quantum digital signature protocol, defined as the loss for which the signal rate is comparable to the dark count rate of the detectors.

## Introduction

The widespread adoption of computers and the internet among global society, and the continuing increase in the number of digital payments, along with the widespread adoption of digital broadcasts, means that there are now at least several exabytes of digital information transmitted each day^1, 2^. A significant proportion of this data is secured using some form of digital signature, which ensures that a malicious party has not tampered with the data in transit, that a legitimate receiver can validate the identity of the signer and that the data is transferable to a third-party who will also accept the message as valid^3–5^. Digital signatures have become so widely used in electronic communications that in 2014 the Council of the European Union passed legislation granting digital signatures the same legal standing as pen and ink handwritten signatures^6^. However, these digital signature schemes are typically based on assumptions regarding the present and future computational difficulty of solving certain problems, such as prime factorisation and finding discrete logarithms^3–5^. There is currently no proof of the computational complexity of these mathematical processes, and it is known that such algorithms are vulnerable to quantum computational algorithms^7^.

This uncertainty regarding long-term security has lead to ongoing research into digital signature schemes that offer security against attackers with unlimited computational capabilities. Two-party message authentication can be achieved with information-theoretic security using schemes such as Wegman-Carter authentication. However, since both participants use the same key for authenticating and verifying messages, it does not provide security against repudiation as a dishonest sender could always claim the message was sent by the other party with access to the secret key. To ensure non-repudiation and transferability, quantum digital signature (QDS) protocols distribute only partial information on the secret key, and does so in such a way that each recipient receives a different verification key. This restriction to partial information is what guarantees that only the sender could have produced the message-signature pair, as only the sender has full information on the key. Although no information-theoretically secure signature scheme can offer universal verifiability, in some cases where it is undesirable to rely on computational security, quantum digital signatures could offer an alternative solution. The optical systems required to implement QDS are similar to those required for quantum key distribution (QKD), and it may be that both QDS and QKD schemes can operate in parallel along the same optical fibers using the same transmitting and receiving hardware. Nevertheless, the underlying protocols are different and they offer complementary services.

Quantum digital signatures were first proposed^8^ in 2001 (and patented^9^ in 2002) but the original protocol required long-term quantum memory as well as a quantum mechanical swap test that made it particularly challenging to implement experimentally. A new protocol that removed the requirement for a swap test was reported^10^ in 2006 and subsequently experimentally demonstrated^11^ in 2012. A typical signature scheme consists of two parts: a signature generation (or distribution) stage and a separate non-interactive messaging stage, that can ideally take place at any time after the distribution stage. By “non-interactive” we mean that a message recipient does not need to communicate with other potential recipients in order to validate a message received from the sender, i.e. verification can be performed locally by the recipient. This first experimental demonstration required that the message either be sent immediately or that a receiver have some form of quantum memory to store the received phase-encoded coherent states sent during the distribution stage until they could be compared to the signature of the message during the messaging stage. Due to the relative immaturity of quantum memory^12^ the experimental demonstration of the 2006 protocol performed the messaging stage immediately after the distribution stage, so that the states sent did not need to be stored.

It was in 2014 that this requirement for quantum memory was lifted by a revision to the protocol^13^. A subsequent experimental demonstration^14^ employed unambiguous state elimination to store classical partial information about the transmitted phase-encoded coherent state. While more practical, this second experimental demonstration still relied on an optical fiber multiport that was composed of two interwoven interferometers closely tying the receivers together, and was therefore hard to scale much above the ≈5 metre separation between receivers demonstrated in the laboratory. Further revisions to the protocol^15^ removed the requirement of an optical multiport by introducing a classical post-processing step performed by the recipients which reproduced the action of the multiport over a single communications link. An experimental demonstration^16^ using a variation of this scheme was able to successfully transmit signatures over 2 km of optical fiber in a laboratory environment. The first demonstration of QDS over a free-space link was conducted over 1.6 km in an urban environment^17^ using a continuous-variable free-space QKD implementation^18, 19^ as the underlying system. The first experimental demonstration of QDS over installed optical fiber^20^ was only conducted over one optical fiber transmission channel at a fixed distance of 90 km. Here, additional optical attenuation (in the form of a variable ND filter) has been combined with a fixed fiber length of 90 km to simulate extended transmission distances permitting operation to be demonstrated over a channel loss corresponding to 134 ± 3.8 km (assuming an overall 0.32 dB/km channel loss as exhibited by the installed optical fiber component) – the longest equivalent distance over which QDS has been shown to operate to date.

The following sections of this paper will introduce the *Methods* (including the *General protocol*, the *Theoretical analysis* and a description of the *Experimental system*) before presenting and discussing the results.

## Methods

### General protocol

The protocol employed in the work reported here is based on our earlier work^21^, but is modified to use differential phase shift (DPS) QKD rather than the decoy state BB84 protocol of the original proposal. Unlike many other quantum communications protocols, here Bob and Charlie are the source of the photons and Alice is the receiver. Alice remains, however, the signatory and the source of the message. Exact definitions of security for unconditionally secure signature protocols are given by Arrazola *et al*.^22^ and a more detailed overview of the security analysis performed for this work is given in the *Theoretical analysis* subsection.

A typical signature scheme consists of two parts: a signature generation (or distribution) stage and a separate messaging stage. Below we outline these stages for the protocol employed here.

#### Distribution

Here a message, *m*, consisting of one bit is considered. Unlike in QKD where one optical pulse is used to secure one bit (after post-processing), QDS uses many pulses to secure a single bit.

- Bob and Charlie independently choose two random sequences of bits, one sequence for each possible one-bit message *m*, either 0 or 1.
- Bob and Charlie both independently carry out two partial QKD^23^ procedures with Alice. By partial, we mean that they proceed only so far as to generate the sifted key and do not proceed to error correction and privacy amplification. They do this separately for *m* = 0 and one for *m* = 1.
- Bob and Charlie send states until Alice has received *L* + *k* successful measurement outcomes. All bits held by Bob and Charlie corresponding to unsuccessful measurements are discarded.
- Bob independently and randomly selects a small number, *k*, of the bits in the key he shares with Alice. Together Alice and Bob sacrifice this part of the key to estimate the error rate between their strings through communication over a classical channel, leaving a remaining key of length *L*. Charlie also undertakes the same procedure with Alice. The remaining *L* measurement outcomes held by Bob and Charlie, corresponding to successful measurement outcomes not sacrificed in parameter estimation, are denoted *B* ^*m*^ and *C* ^*m*^ respectively.
- Bob and Charlie randomly and independently split their measurement outcomes into two sets, each containing *L*/2 measurement outcomes. We call these sets ${B}_{1}^{m}$, ${B}_{2}^{m}$, ${C}_{1}^{m}$ and ${C}_{2}^{m}$. They each forward the set indexed “2” to the other using a standard QKD^24^ link (i.e. including error correction and privacy amplification). Bob and Charlie keep secret from Alice the bits that are forwarded and the bits that are retained.

At the end of this process, Alice holds information correlated with the strings *B* ^*m*^ and *C* ^*m*^. However, she has no information as to whether each element in *B* ^*m*^ and *C* ^*m*^ is held by Bob or Charlie following the symmetrization procedure they perform in step 5 of the distribution stage. On the other hand, Bob has full information on ${B}_{1}^{m}$ and ${C}_{2}^{m}$, but has no information on ${C}_{1}^{m}$. Similarly Charlie has full information on ${C}_{1}^{m}$ and ${B}_{2}^{m}$, but not ${B}_{1}^{m}$.

#### Messaging

All communication during the messaging stage takes place over pairwise authenticated classical communication channels; quantum communication is needed only in the distribution stage. Here we will assume that Alice sends a single-bit message *m* to Bob who then wishes to forward it to Charlie. The choice of recipient is arbitrary, and the protocol would work in the same manner if Alice chose to send the message to Charlie instead.

- Alice sends the message *m* to Bob together with her corresponding signature, which is comprised of the 2*L* measurement outcomes from the states sent to her by each of Bob and Charlie for the corresponding *m*.
- Bob checks Alice’s *L* bit signature separately against both ${B}_{1}^{m}$ and ${C}_{2}^{m}$. He accepts the message if he finds fewer than *s*_*a*_ · *L*/2 mismatches with both ${B}_{1}^{m}$ and ${C}_{2}^{m}$, where *s*_*a*_ is an authentication threshold. Otherwise, he rejects the message.
- To forward the message to Charlie, Bob transmits *m* to him along with Alice’s 2*L* measurement outcomes.
- Charlie checks the signature against the bits he received from Bob, and against the states he sent to Alice and did not forward to Bob, for message *m*. He accepts the message if he finds fewer than *s*_*v*_ · *L*/2 mismatches with both ${C}_{1}^{m}$ and ${B}_{2}^{m}$, where *s*_*v*_ is an authentication threshold chosen such that *s*_*v*_ > *s*_*a*_. Otherwise, he rejects it.

Signing a message uses up the distributed signature, which cannot be reused. It is important that *s*_*a*_ < *s*_*v*_, i.e. that the threshold for accepting a message directly from Alice is strictly less than the threshold for accepting a forwarded message, otherwise Alice could repudiate with high probability. The parameters *s*_*a*_ and *s*_*v*_ will be defined in greater detail in the following section. We also note that techniques exist^25^ to leverage our single-bit signing scheme into one which can sign longer messages. Alternatively, the scheme can simply be iterated to sign each bit of a longer message individually. However, in the latter case security needs to be carefully defined and may be application specific.

### Theoretical analysis

In this section we consider the theoretical analysis of the security parameters that allow us to calculate the authentication threshold *s*_*a*_ and verification threshold *s*_*v*_ and subsequently the length *L* of the bit sequence required to sign a single-bit message *m* = 0 or 1 to a security level of *ε*. This section will provide a general overview of the security considerations and calculations. Since the distribution stage outlined previously in the *General protocol* section takes place over quantum channels, quantum effects must be analyzed in order to quantify the information obtainable by an eavesdropper when considering the security of the protocol. If we are to consider this protocol as secure then we require that the following conditions are met^26^:

1. Robustness: If all participants are honest, the recipients will accept valid messages and the protocol does not abort.
2. Unforgeability: Except with negligible probability, it should not be possible for an adversary to create a valid signature.
3. Non-repudiation: Except with negligible probability, a signer should be unable to repudiate a legitimate signature that he has created.
4. Transferability: If a verifier accepts a signature, he should be confident that any other verifier would also accept the signature.

It is important to stress that for transferability, a recipient should be able to test whether other recipients are likely to accept the message without contacting other potential recipients in the messaging stage. In the three-party case considered here, security against repudiation implies that the message is transferable if a majority vote is used to resolve disputes.

The security analysis presented here is restricted to adversaries capable of performing only independent attacks and sequential attacks, which are the most realistic attacks given current technology. It builds on the work carried out by Diamanti^27^ to bound the success probability of an eavesdropper forging a message. Further, security in the completely general setting could be proven using the results of Wen *et al*.^28^ and Tamaki *et al*.^29^, but would require a slightly modified experimental set-up as well as photon-number-resolving detectors. Photon-number-resolving detectors are experimentally challenging to implement and are presently impractical for deployed systems.

The security analysis first considers the probability of Eve incorrectly guessing the bit sent by Bob, which is computed^27^ as,

1 $$\begin{array}{ccc}{P}_{e} & = & 1-\,max\{2{|\alpha |}^{2}(1-\beta )+[1-2{|\alpha |}^{2}(1-\beta )]\times \,[1-{{\rm{Q}}{\rm{B}}{\rm{E}}{\rm{R}}}^{2}-\frac{1}{2}{(1-6\cdot {\rm{Q}}{\rm{B}}{\rm{E}}{\rm{R}})}^{2}],\\  &  & 2d\cdot {\rm{Q}}{\rm{B}}{\rm{E}}{\rm{R}}+\,\frac{1}{2}(1-2d\cdot {\rm{Q}}{\rm{B}}{\rm{E}}{\rm{R}})\},\end{array}$$

where *β* is the total transmission efficiency of the system, QBER is the quantum bit error rate^30^, |*α*|^2^ is the mean photon number per pulse^27^, and

2 $$d={\mathrm{log}}_{{|\alpha |}^{2}}(\beta +1).$$

Hoeffding’s inequalities^31^ are then used, together with the methods in our previous work^21^, to find the probabilities of an accidental abort under honest conditions,

3 $$P({\rm{Honest}}\,{\rm{Abort}})\le 2\,\exp \,[-{({s}_{a}-{\rm{QBER}})}^{2}L],$$

the probability of repudiation of a valid message,

4 $$P({\rm{Repudiation}})\le 2\,\exp \,[-{(\frac{{s}_{a}-{s}_{v}}{2})}^{2}L],$$

and the probability of successful forging,

5 $$P({\rm{Forge}})\le 2\,\exp \,[-{({P}_{e}-{s}_{a})}^{2}L].$$

We call the system secure to the level *ε* if all of the probabilities presented in 3, 4, and 5 are smaller than *ε*. That is, for the protocol to be secure, we require that

6 $$\varepsilon \ge \,{\rm{\max }}\,[P({\rm{Forge}}),P({\rm{Repudiation}}),P({\rm{Honest}}\,{\rm{Abort}})].$$

Since there is no reason to favor one probability over any of the others for the system reported here, we set the *s*_*a*_ and *s*_*v*_ parameters to be

7 $${s}_{a}={\rm{Q}}{\rm{B}}{\rm{E}}{\rm{R}}+\frac{{{\rm{P}}}_{{\rm{e}}}-{\rm{Q}}{\rm{B}}{\rm{E}}{\rm{R}}}{4}$$

and

8 $${s}_{v}={\rm{Q}}{\rm{B}}{\rm{E}}{\rm{R}}+\frac{3({{\rm{P}}}_{{\rm{e}}}-{\rm{Q}}{\rm{B}}{\rm{E}}{\rm{R}})}{4}$$

so that

9 $$P({\rm{Forge}})=P({\rm{Repudiation}})=P({\rm{Honest}}\,{\rm{Abort}}).$$

**Figure 1:**
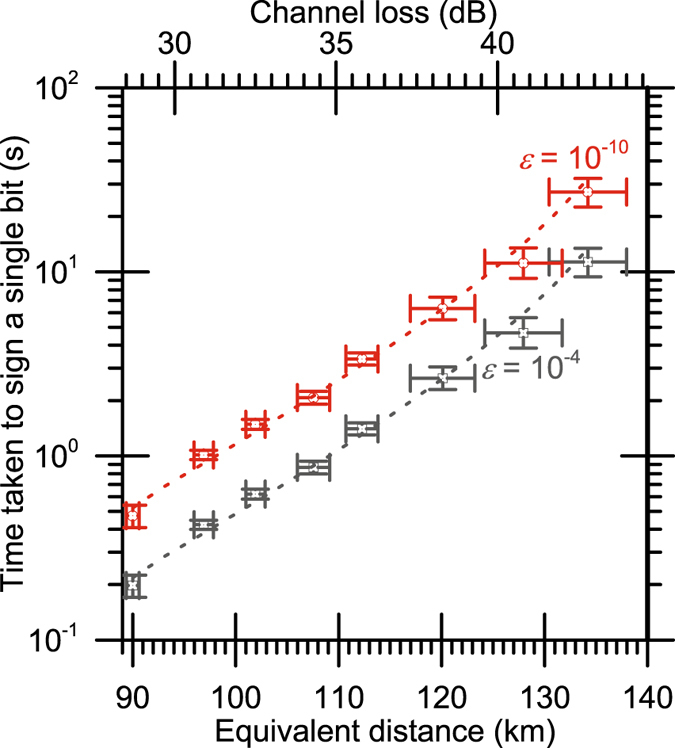
The variation in time taken to sign a single message bit with the transmission loss of the quantum channel, as also presented in Table 1. The gray data points present a security level *ε* of 10^−4^, as used in our previous demonstrations of QDS. The red data points present a security level *ε* of 10^−10^, as used in many QKD experiments. Dashed lines indicate theoretical predictions from a model of the system^36^. Equivalent distances have been calculated from the additional attenuation using the 0.32 db/km unit loss of the installed 90 km optical fiber link.

For the results presented in Table 1 and Fig. 1, the signature length *L* has been calculated from 3, 4, and 5 and used in conjunction with the count rate at receiver Alice to determine the time required to sign a single bit (i.e. the time required to send sufficient coherent states to give Alice the ability to sign either *m* = 0 and *m* = 1).

**Table 1:** The variation in time taken to sign a single message bit with the transmission loss of the quantum channel, as also presented in Fig. 1.

Channel loss		Equivalent distance		QBER		*ε* = 10^−4^		*ε* = 10^−10^	
						Time to sign a bit		Time to sign a bit	
(dB)	(±)	(km)	(±)	(%)	(±)	(s)	(+/−)	(s)	(+/−)
28.7^†^	0.2	90.0	0.6	0.93	0.37	0.2	0.03/0.03	0.47	0.07/0.07
30.9	0.3	96.9	0.9	1.22	0.15	0.42	0.02/0.02	1.01	0.06/0.06
32.5	0.3	101.9	0.9	1.21	0.15	0.62	0.04/0.04	1.49	0.09/0.09
34.3	0.5	107.6	1.6	1.38	0.16	0.87	0.07/0.07	2.07	0.17/0.16
35.8	0.5	112.3	1.6	1.43	0.10	1.41	0.11/0.10	3.37	0.26/0.25
38.3	1.0	120.1	3.1	1.62	0.20	2.65	0.4/0.35	6.34	0.96/0.84
40.8	1.2	127.9	3.8	1.79	0.25	4.66	0.97/0.81	11.17	2.33/1.95
42.8	1.2	134.2	3.8	2.75	0.28	11.33	2.11/1.93	27.13	5.04/4.61

The attenuation of 28.7 ± 0.2 dB marked with a † represents the attenuation of the fixed 90 km of installed optical fiber. Equivalent distances have been calculated from the additional attenuation using the 0.32 db/km unit loss of the installed 90 km optical fiber link.

### Experimental system

**Figure 2:**
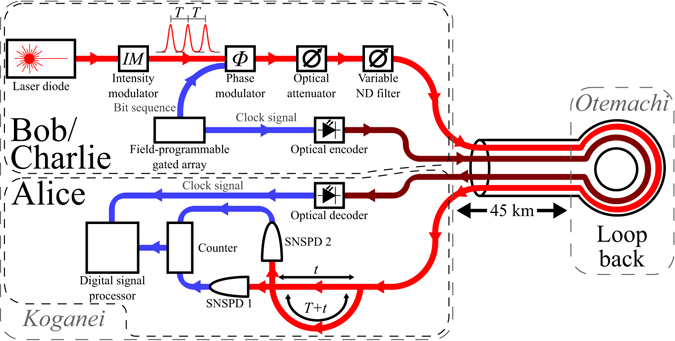
The underlying QKD system that underpins this QDS experiment is based on a DPS system developed by NTT^33^. The channel between senders Bob/Charlie and receiver Alice was composed of 45 km of installed optical fiber in a loop-back configuration between NICT laboratories at Koganei and Otematchi to give a total optical path length of 90 km, and additional attenuation introduced by a variable ND filter.

The goal of the work reported here was to demonstrate that the generation of QDS can be conducted over installed optical fiber using QKD systems. To that end, the experimental system used for the QDS demonstration reported here, shown in Fig. 2, was based on a DPS-QKD system developed by the Nippon Telegraph and Telephone Corporation (NTT) for use in the Tokyo QKD network^32, 33^. DPS-QKD offers advantages over BB84-type protocols in that there is no requirement to carry out basis set reconciliation process and bit rates can potentially be higher. However, the security analysis of such a system^27–29^ is less comprehensive than for BB84-type systems^34^. In accordance with the QDS protocol, Bob and Charlie were operated as transmitters of photons and Alice was a receiver^21^. However, Alice remains the signatory of any messages. One fiber link and one DPS-QKD system was employed for this demonstration, with the transmitter first operating as Bob and then subsequently Charlie in a time-multiplexed configuration. The sender and receiver were located in the NICT laboratories at Koganei, Tokyo, Japan.

The transmitting Bob/Charlie system used a continuous wave (CW) laser diode with a central wavelength of 1551 nm as the source of the coherent states. The CW output was modulated into a series of pulses using a lithium niobate (LiNbO_3_) optical intensity modulator driven at clock rate of 1 GHz so that the time between the center of each optical pulse, *T*, was equal to 1 ns. For each optical pulse, a field programmable gated array (FPGA) selected a phase of 0 or *π* radians which was subsequently imparted on the optical signal by a LiNbO_3_ phase modulator. Following this, the intensity of the optical pulses, |*α*|^2^, was attenuated to a mean photon number per pulse of 0.2. Therefore, the probability amplitude of a single photon is spread into five subsequent pulses and the probability of two or more photons existing in a single pulse is reduced to 0.016 for all pulses (including vacuum), down from 0.184 for a mean photon number of 1 photon per pulse. Before emission from Alice in the 90 km installed optical fiber link, the pulses were further attenuated by means of a variable neutral-density (ND) filter to simulate additional lengths of optical fiber by way of additional attenuation. A 100 kHz clock rate optical synchronization signal at a wavelength of 1560 nm was provided by a bright (i.e. multi-photon) pulse from an optical encoder.

The installed optical fiber link was comprised of a fixed 90 km of 9 *μ*m core diameter standard telecommunications optical fiber in a 45 km loop-back configuration from Koganei, via Otemachi, back to Koganei. The optical fiber link was installed in both underground ducting and overhead poles, with approximately half of the total length in each situation. The installed optical fiber link was ‘dark’ in the sense that the photons associated with the QDS communications were the only signals intentionally propagated through the channel. Additional attenuation was introduced via a variable neutral-density (ND) filter at Bob/Charlie to simulate longer transmission distances. The complete round-trip of photons in the 90 km optical fiber channel had a loss of 28.7 ± 0.2 dB, giving a per-unit length loss of 0.32 dB/km, used in conversion of the additional attenuation into equivalent length.

Receiver Alice employed a temperature-stabilized silica planar light-wave circuit to introduce a delay of *T* = 1 ns such that an incident optical pulse could be interfered with the next pulse. The interference at the lightwave circuit had a visibility of 98%. The phase difference between the two successive pulses, which was either 0 or *π*, determined which superconducting niobium nitride superconducting nanowire single-photon detector (SNSPD) the pulse was routed towards for detection. One detector was denoted as signifying a binary 0 while the other denoted a binary 1. At an operating temperature of 2.5 K, the SNSPDs exhibited a dark count rate *D*_*c*_ of less than 100 counts per second and a mean detection efficiency *η* of 20%. An optical decoder received the synchronization signal for Alice and an optical band-pass filter (BPF) with a 1.5 dB loss was inserted at the receiver end of the quantum channel to suppress the clock signal and wideband background noise.

The classical data exchange was carried out using an Ethernet^35^ link between Alice and Bob/Charlie.

### Data Availability

All data associated with this project may be downloaded from the Heriot-Watt University data archive at doi:10.17861/7c97c071-02b6-45de-b074-d96c87e9e207.

## Results and Discussion

The results for the system can be seen in Table 1 and Fig. 1. Equivalent distances have been calculated from the additional attenuation using the 0.32 db/km unit loss of the installed 90 km optical fiber link. The quoted uncertainty in the QBER was calculated by subdividing the single-photon detector events into a series of blocks of 10,000 bits each and computing the standard deviation of the QBERs from each of the blocks. The system reported here represents a significant advance in the operating length of QDS systems.

The security level *ε* represents the maximum probability of the protocol failing. Hence, except with probability *ε*, the protocol will not abort unnecessarily and dishonest parties will not be able to forge, repudiate, or create non-transferable messages. Successful operation has been shown over a combination of installed optical fiber and additional channel loss corresponding to 134.2 ± 3.8 km of installed optical fiber at two security levels, *ε* = 10^−4^, as used in many previous demonstrations of QDS^11, 14, 16, 17^ and *ε* = 10^−10^, as commonly used in QKD systems. The performance of this system is enhanced compared to that reported previously over 90 km of installed optical fiber^20^ in that it can now sign approximately 5 bits per second at 90 km for an *ε* of 10^−4^, as opposed to 2 bits per second previously, and 2 bits per second at an *ε* of 10^−10^, as opposed to 1. This is due to optimization of the bias voltage on the superconducting nanowire detectors. At a security level *ε* of 10^−4^ our previous system^16^ took 20 s to sign a single bit when operating under optimal conditions over 500 m of fiber in the laboratory. For the same security level, the system reported here can sign two bits in 20 s over a channel loss corresponding to an operating distance of 134.2 ± 3.8 km or two bits per second at a security level of 10^−10^ over a channel loss corresponding to 127.9 ± 3.8 km. The largest channel loss of 42.8 ± 1.2 dB, corresponding to an optical fiber length of 134.2 ± 3.8 km, was the highest loss that the experiment could be conducted over. In order to undertake a statistically significant finite key-size analysis at this channel loss, a data acquisition duration in excess of 3 hours was required. In effect, this means that with this protocol, it is unlikely that we can expect to operate over practical timescales with any higher loss than that demonstrated in this paper. Extrapolation of a theoretical model based on previous analysis of QKD systems^36^ and the known parameters of this system^33^ indicates that the absolute maximum operating channel loss for this system is around 50 dB (or a fiber length of around 156 km), at which level the sifted key rate becomes comparable to the dark count rate of the detectors. Other QDS^10, 13^ protocols are unlikely to operate at a higher channel loss, and require significantly less channel loss to operate over practical timescales. Recent experiments targeting the application of a decoy state BB84 protocol QKD to satellites^37^ have been able to successfully generate keys over channel losses of up to 56.5 dB. There is no reason to believe that such satellite based QKD systems could not be used for QDS to provide a means of transmitting signatures over longer ranges.

The work reported here has experimentally demonstrated that optical fiber QKD systems can be operated over long distances to provide the additional functionality of QDS. By employing an existing QKD system over such long distances, this work indicates that QDS has moved beyond the realm of simple laboratory-based demonstration test-bed systems and is now potentially ready for practical deployment alongside QKD systems. Although this system used a DPS-QKD demonstrator as the underlying optical system, there is no reason to believe that the performance of a phase-encoded BB84 type system would be significantly different in terms of time to sign bits at these levels of channel loss. By demonstrating that QDS protocols can be applied to an alternative QKD protocol, other than BB84, this work has shown that there is scope for use of QDS with further alternative QKD systems such as coherent-one-way^38^ or sub-carrier wave^39^. With revised protocols for signatures offering the possibility of even greater enhancements in signature generation rate^40^, the prospect of commercial systems capable of offering both QKD and QDS, depending on the end application required, is potentially close and recent developments in chip scale QKD systems^41^ also offer the prospect of compact chip scale QDS systems. QDS could be used to secure long-term data storage in future distributed backup and access solutions^42^.
